# Quality of Life, Disability, Well-Being, and Coping Strategies in Patients Undergoing Neurosurgical Procedures: Preoperative Results in an Italian Sample

**DOI:** 10.1155/2014/790387

**Published:** 2014-11-03

**Authors:** Silvia Schiavolin, Rui Quintas, Marco Pagani, Stefano Brock, Francesco Acerbi, Sergio Visintini, Alberto Cusin, Marco Schiariti, Morgan Broggi, Paolo Ferroli, Matilde Leonardi

**Affiliations:** ^1^Neurology, Public Health and Disability Unit, Neurological Institute C. Besta IRCCS Foundation, Via Celoria 11, 20133 Milan, Italy; ^2^Division of Neurosurgery II, Neurological Institute C. Besta IRCCS Foundation, Via Celoria 11, 20133 Milan, Italy

## Abstract

*Background*. The aim of this paper is to present the preliminary results of QoL, well-being, disability, and coping strategies of patients before neurosurgical procedure. *Methods*. We analysed data on preoperative quality of life (EUROHIS-QoL), disability (WHODAS-II), well-being (PGWB-S), coping strategies (Brief COPE), and functional status (KPS score) of a sample of patients with brain tumours and cerebrovascular and spinal degenerative disease admitted to Neurological Institute Carlo Besta. Statistical analysis was performed to illustrate the distribution of sociodemographic and clinical data, to compare mean test scores to the respective normative samples, and to investigate the differences between diagnoses, the correlation between tests, and the predictive power of sociodemographic and clinical variables of QoL. *Results*. 198 patients were included in the study. PGWB-S and EUROHIS-QoL scores were significantly lower than normative population. Patients with spinal diseases reported higher scores in WHODAS-II compared with oncological and cerebrovascular groups. Finally sociodemographic and clinical variables were significant predictors of EUROHIS-QoL, in particular PGWB-S and WHODAS-II. *Conclusion*. Our preliminary results show that preoperatory period is critical and the evaluation of coping strategies, quality of life, disability, and well-being is useful to plan tailored intervention and for a better management of each patient.

## 1. Introduction

Health related quality of life (HRQoL) has become an important measure in clinical trials in patients with primary brain tumours [1], cerebrovascular disease [2], and spinal disease [3]. HRQoL, assessed using self-reported questionnaires, is defined as a personal self-assessed ability to function in the physical, psychological, emotional, and social domains of day-to-day life and reflects an individual's overall satisfaction with life [4–6]. The evaluation of patients' quality of life (QoL) provides new and important information that could be used by clinicians, researchers, and patients guiding treatment decisions and prognosis [7]. HRQoL data can be also a critical factor in understanding new treatments, not simply in terms of traditional endpoints, such as survival, but also in terms of understanding what that additional survival means to each patient [7–9].

Disability and psychological well-being (PWB) are also significant factors in the multidimensional assessment of patients: disability is considered the complex interaction between a person's health condition and contextual factors according to biopsychosocial approach of International Classification of Functioning, Disability, and Health (ICF) [10], while PWB is the measure of self-representations of intrapersonal affective or emotional states reflecting a sense of subjective well-being or distress [11]. Finally, the investigation of coping strategies—that is, the ability to face difficult situations—is important because they can attenuate the relationship between undesirable life events and personal functioning [12].

Although patient's prospective can be very useful in the medical decision-making process, studies usually concentrate on medical perspective reporting morbidity and mortality and forget the biopsychosocial approach that should be patient oriented. In the process of getting informed consent before surgery, as also reported by Neil-Dwyer et al., patients should be informed not only on pure surgical effects of intervention but also on possible effects on QoL, PWB, and functioning [13]. Few studies have addressed these indices in the brain tumour surgery [13–17], where the evaluation of HRQoL is also important because patients with brain tumour may suffer from a wide range of neurological deficits, cognitive dysfunctions, and personality changes that are related to the brain disease or to the effect of neurosurgical treatment and that can impact on QoL and PWB [18]. Most studies about this issue focused indeed on patients with brain tumour in general [1, 19–21]. Regarding spinal surgery, studies on disability and QoL are instead frequent enough [22–25].

Finally, most of studies focus on a specific disease and do not compare QoL, disability, and PWB among different neurosurgical pathologies [16].

An observational longitudinal study on neurosurgical complications and preoperative and postoperative QoL, PWB, disability, and coping strategies of patients undergoing neurosurgical procedure is performed since January 2012. The aim of this paper is to present a description of preoperative QoL, PWB, disability, and coping strategies of a sample of patients of this study with oncological (ONC), cerebrovascular (CV), and spinal degenerative (SD) diseases. Furthermore we analysed the relationship between these parameters and compared them in diagnosis.

## 2. Materials and Methods

We selected a sample of patients among people admitted to Neurosurgery Unit II at Neurological Institute Carlo Besta IRCCS Foundation from May 2012 to June 2013. We did not enroll people that were admitted to the hospital on the same day of surgery, that refused to participate in this study, that were with an age < 18, that were with recurring brain tumours, that did not have a good Italian comprehension, or that were difficult to involve because of the lack of time due to medical exams being already planned. Among people enrolled, we excluded patients with severe cognitive problems (MOCA < 19) or incomplete test.

The study was approved by ethics committee and a psychologist provided the informed consent and collected data on preoperative QoL, disability, PWB, and coping strategies.

Patients included in this study underwent neurosurgical procedure for a variety of CV diseases (such as aneurysm, cavernous hemangioma, arteriovenous malformations, and ischemic cerebral disease requiring bypass procedure), ONC diseases (such as adenomas, craniopharyngioma, meningioma, gliomas, and neurinomas), and SD diseases (such as discal hernia, stenosis, and spinal instability).

This paper presents the preliminary results of QoL, disability, PWB, and coping strategies before neurosurgical procedure of a sample of adult patients, analyses the relationship between these parameters, and compares them among patients with ONC, CV, and SD diseases.

### 2.1. Assessment Protocol

The assessment protocol was composed of sociodemographic schedule and self-report questionnaires.

EUROHIS-QoL is a measure for QoL derived from the WHO-QoL (WHO Quality of Life Instrument) and is composed of 8 items based on a five-step scale. The sum of scores on the eight items forms the overall QoL: higher scores indicate better QoL [26]. For our analysis mean scores obtained in EUROHIS-QoL instrument were used.

WHODAS-II (WHO Disability Assessment Schedule, second version) is composed of 12 items and assesses disability taking into account patient's difficulties in performing different activities caused by health condition. WHODAS-II covers six domains: cognition, mobility, self-care, getting along, life activities, and participation. Items are based on a scale of 1–5 and the overall score ranges from 0 to 100 with higher scores indicating higher disability levels [27].

PGWB-S (Psychological General Well-Being Index-Short) is the 5-item version of the original PGWB to investigate self-perceived psychological PWB through the assessment of mental health and perceived vitality status. The overall score is formed by the sum of scores on the five items based on a six-step scale: higher scores indicate higher level of PWB. The scores are later transformed to obtain scores ranging from 0 to 110 comparable with the longer version [28].

Brief COPE assesses different coping behaviours and thoughts that people may have in a stress condition. It is composed of 28 items composing 14 subscales: self-distraction, active coping, denial, substance use, use of emotional support, use of instrumental support, behavioural disengagement, venting, positive reframing, planning, humour, acceptance, religion, and self-blame. Items are based on a scale of 1–4 and single scores were then codified for composing the scales' scores ranging from 0 to 8 where higher scores indicate higher usability of the coping strategy [29].

Patients were selected using MOCA (Montreal Cognitive Assessment) test: scores lower than 19, indicating the presence of a cognitive impairment, were used as cut-off for the inclusion in the study. MOCA is a 30-point test investigating the following areas: short-term memory recall, delayed recall after approximately 5 minutes, visuospatial abilities, executive functions, attention, concentration and working memory, language, and orientation to time and place [30].

For the research results presented in this paper, diagnosis and Karnofsky Performance Status Scale (KPS) score were also taken into consideration. Patients were categorized into five groups based on the diagnosis: ONC, CV, SD, oncological spinal (OS), and a further group containing another diagnosis (such as hydrocephaly, Arnold-Chiari malformation).

KPS is a clinical score obtained from a numerical scale from 0 to 100 representing a patient's ability to perform daily and working activities, self-care, and the need for assistance. Higher scores suggest a better functional status [31].

### 2.2. Statistical Analysis

Descriptive statistics were conducted to illustrate the distribution of sociodemographic and clinical variables, as well as PGWB-S, EUROHIS-QoL, and WHODAS-II scores and KPS.

One-sample t-tests were calculated to compare PGWB-S and EUROHIS-QoL mean scores of my sample with PGWB-S and EUROHIS-QoL mean scores of the respective normative samples. As normative scores of PGWB-S are reported in the literature divided by age group, a weighted score was calculated to have a unique mean value with the following formula: unique overall normative score = normative score for each age categories ∗ number of participants for that age category/total number of participants. One-sample *t*-test was also used to compare WHODAS-II mean scores with the values of other clinical populations since a normative value is not reported in the literature. For Brief COPE, most frequent coping strategies were defined when scores were 6, 7, or 8.

Pearson's product-moment coefficient was calculated to assess bivariate correlations of PGWB-S, EUROHIS-QoL, and WHODAS-II both in whole sample and in three specific groups: ONC, SD, and CV. The bivariate correlation between KPS and EUROHIS-QoL was also calculated in whole sample with Pearson's product-moment coefficient. Correlations were considered weak with coefficient values < .29, moderate with values between .30 and .59, and strong with values > .60 [32]. Bonferroni adjustment was applied to multiple comparisons to reduce type 1 error.

Comparisons of KPS, PGWB-S, EUROHIS-QoL, and WHODAS-II scores in the three groups—ONC, SD, and CV—were evaluated using nonparametric Kruskal-Wallis statistics and post hoc *t*-tests corrected with Bonferroni's procedure were calculated for pair-wise comparison (OS and the other group were excluded from this analysis due to the reduced number of patients: 7 and 6 patients, resp.).

A multiple regression analysis was performed to evaluate the extent to which KPS, diagnosis, PGWB-S, WHODAS-II, and sociodemographic variables such as gender, years of study, and age have predictive power on EUROHIS-QoL. The multiple regression analysis was performed with both the whole sample and ONC, SD, and CV groups (OS and the other group were also excluded from this analysis due to the reduced number of patients).

All statistical analyses were performed using SPSS v. 18.0.

## 3. Results

### 3.1. Results of the Whole Sample

From January 2012, 909 people were admitted to the hospital. On the basis of the enrolment criteria, we involved in our study 234 patients undergoing neurosurgical procedures from May 2012 to June 2013. Of this sample, 36 patients were excluded due to the presence of cognitive problems or incomplete test. 198 patients were considered: 103 (48%) females, with a mean age of 51 (SD = 13.5; range = 19–83) and mean of years of study of 12.4 (SD = 4.25). 42 (21.2%) were patients with CV diseases, 89 (45%) with ONC diseases, 54 (27.3%) with SD diseases, 7 (3.5%) with OS, and 6 (3%) with other diseases. The last two groups were not considered for ANOVA between diagnosis and multiple regression analysis (Table 1).

Mean KPS score was 95.71 (SD = 7.95; range = 40–100); 136 (68.7%) participants had a KPS of 100, 47 (23.7%) of 90, and 15 (7.6%) of 80 or lower. Detailed scores obtained at tests are reported in Table 2.

Comparisons between PGWB-S and EUROHIS-QoL and respective normative scores showed PGWB-S and EUROHIS-QoL scores significantly lower than the ones obtained by normative population (Table 3). Additionally, mean score of WHODAS-II is similar to the scores obtained by stroke patients (25.9; *P* = 0.937) and higher than the one from patients with epilepsy (13.6; *P* < 0.001) [33, 34].

Regarding coping strategies used by our sample, more than 35% of the sample indicated planning (45.96%), acceptance (42.93%), self-distraction (39.90%), and positive reframing (38.38%) as the most frequently used coping strategies (scoring 6 or higher), whereas substance use (2.02%), denial (5.05%), self-blame (10.61%), and use of emotional support (12.63%) were the less adopted strategies (Table 4).

Correlations between tests were performed: EUROHIS-QoL and PGWB-S (*r* = 0.549^**^; *P* = 0.000), EUROHIS-QoL and WHODAS-II (*r* = −0.504^**^; *P* = 0.000), and PGWB-S and WHODAS-II (*r* = −0.523^**^; *P* = 0.000) were significantly and strongly correlated considering the whole sample. Similar results and coefficients were found when the analysis was calculated by groups (ONC, CV, and SD). Finally KPS and EUROHIS-QoL were significantly but weakly correlated (*r* = 0.178; *P* = 0.012).

### 3.2. Results of ONC, CV, and SD Groups

Table 5 shows differences based on the diagnosis, which were statistically significant for KPS and WHODAS-II. Patients with SD diseases reported higher scores in WHODAS-II when compared to ONC group (nonparametric post hoc *t*-test = −2.998; *P* = 0.003) and CV group (nonparametric post hoc *t*-test = −3.591; *P* = 0.000), while CV group had KPS score higher than ONC group (nonparametric post hoc *t*-test = −2.627; *P* = 0.009). No differences were detected in the EUROHIS-QoL and PGWB-S.

### 3.3. Regression Analysis

Altogether, gender, years of study, age, KPS, diagnosis, PGWB-S, and WHODAS-II were significant predictors of EUROHIS-QoL in the whole sample (*R*2 = 0.38; Adj. *R*2 = 0.35; SE = 0.46; *F* = 12,50; *P* < 0.001). In particular, PGWB-S (*P* < 0.001) and WHODAS-II (*P* < 0.007) had a significant relationship (positive and negative, resp.) with EUROHIS-QoL (Table 6). We found similar results both in CV (*R*2 = 0.38; Adj. *R*2 = 0.27; SE = 0.43; *F* = 3.44; *P* = 0.009) and in ONC groups (*R*2 = 0.45; Adj. *R*2 = 0.41; SE = 0.50; *F* = 10.49; *P* = 0.000) with a positive and significant relationship of PGWB-S (*P* = 0.050 in CV group; *P* = 0.001 in ONC group) with EUROHIS-QoL and a negative and significant relationship of WHODAS-II (*P* = 0.031 in CV group; *P* = 0.005 in ONC group) with EUROHIS-QoL (Table 7). In the SD group (*R*2 = 0.29; Adj. *R*2 = 0.19; SE = 0.41; *F* = 2.93; *P* = 0.017) only PGWB-S had a significant and positive relationship (*P* = 0.020) with EUROHIS-QoL (Table 7).

## 4. Discussion

This paper provides a description of QoL, PWB, disability levels, and coping strategies of a selected sample of 198 adult persons undergoing neurosurgical procedure, compares their values with normative populations, and defines the relations between all these parameters in different diseases (ONC, CV, and SD).

The preoperative functional status of our sample measured with KPS scale, independently of diagnosis, showed that majority of patients maintained their ability to perform daily and working activities and self-care and presented lower levels of need for assistance. This result is in line with a study by Palese et al. that reported a good performance status for the majority of brain neoplasm patients before surgery [35]. This result is also similar to a study by Tsay et al. where patients with benign primary brain tumours tended to be independent prior to surgery [17]. However, the absence of severe difficulties in performing daily activities as measured by KPS can be related to the selection bias for which more impaired people were excluded from this study.

QoL and PWB levels in the whole sample were significantly lower than the ones obtained by normative population which is in line with previous works such as Miao et al. and Whittle et al. They reported preoperative HRQoL scores lower than normal population, respectively, in a sample of patients with meningioma and patients with brain tumour and spinal disease [15, 36].

Disability level of our sample was high. If compared with other health conditions, it appeared similar to stroke that is considered a high disabling disease and higher than epilepsy that seemed instead to look like general population [33, 34].

These results were expected: QoL and PWB were strongly and positively correlated constructs and both had a significant and negative correlation with the disability level. Furthermore, QoL and functional status measured by KPS were positively correlated, as reported also in other studies, where KPS generally correlates with overall QoL [37, 38].

We found some outcomes' differences between different diagnoses: patients with spinal diseases had a higher preoperatory disability level when compared to oncological and vascular patients. WHODAS focuses mainly on motor activities that are usually more impaired in spinal patients. SD patients in fact had chronic motor symptoms from a long period while our sample of ONC and CV patients often had a single acute event or, sometimes, the tumour or the vascular problem had been discovered during medical examinations for other reasons. Furthermore due to our exclusion criteria we did not include patients with recurrent tumours or cognitive problems and we got many refusals to participate from patients with more severe symptoms with the consequence which was that we probably excluded more problematic ONC patients. So it is necessary to take into account this selection bias when comparing different diagnoses.

Some differences in KPS scale were found that could be explained by disease clinical characteristics as CV group had a better functional status comparing to ONC group.

No differences were detected in QoL between diagnoses contrary to a study by Whittle et al. where patients with spinal disease were worse than the brain tumour patients in many domains of QoL [36]. This result can be related to the different instruments used in Whittle et al. and our studies. On the other hand, it is possible that different factors and situations determined similar QoL in ONC, CV, and SD groups or the surgical context influenced the responses to tests on QoL in the three groups. Similarly, no differences in PWB in the three different groups were found.

Frequently, our sample used as coping strategies planning, acceptance, self-distraction, and positive reframing in the preoperative period. This result is very similar to the study of Palese et al. where patients with brain neoplasm adopted more optimistic coping strategies and a positive approach towards the situation probably to facilitate the process of adaptation [35].

Finally we found that altogether sociodemographic and clinical variables (gender, years of study, age, KPS, diagnosis, PGWB-S, and WHODAS-II) accounted for the variance in QoL in the whole sample. PWB and disability levels were the only variables significantly related to higher or lower QoL levels, respectively. EUROHIS-QoL is in fact correlated with other measures of mental, physical health and PWB as reported in a study by da Rocha et al. [39]. Furthermore similar results were founded by Bunevicius et al. that reported psychological distress, symptom severity, functional disability, and cognitive impairment as important determinants of poor HRQoL in a sample of patients with brain tumours [40].

Considering the low levels of QoL and PWB shown by our sample when compared to normal population, we can assume that preoperatory period is particularly critical for patients undergoing neurosurgical procedure. In this sense, psychological interventions should be considered to support patients during the preoperative period. The evaluation of coping strategies adopted by each patient and QoL, disability, and PWB indicators are also useful elements to plan tailored intervention that can complement surgical procedures and can respond better to the specific needs of each patient. Trying to understand within a biopsychosocial perspective the individual needs and reactions towards illness and treatments is important for a better pre- as well as postsurgery management of each patient.

The study results should be cautiously generalized to other neurosurgical populations due to the high specialization of the Neurological Institute Carlo Besta, the reduced size of the sample, and the selection bias aforementioned.

Some limitations of our study should be taken into account. First, within each of the three different groups there are several differences, in grading, staging, severity, and anatomical localization, that for the moment we did not take into account but that can make each group very heterogeneous. Future analysis should be made on a larger sample comparing more specific diagnostic groups or dividing and comparing patients based on other clinical variables. A second limitation is related to the timing of performing the interview: patients often filled the protocol the day before surgery, when anxiety and any other psychological distress may act as a bias for the responses. Finally, the exclusion of patients with severe cognitive problems and recurring brain tumours caused the omission of more severe and problematic clinical situations in the ONC group.

Future research on the differences between diagnoses should be performed taking into account the cognitive status. This could be different in patients with ONC, CV, or SD diseases and have an impact on QoL, disability, and PWB. Furthermore all these indices should be also explored and compared between the subpathologies of each diagnostic group.

By the end of the entire project, we will be able to compare data on QoL, disability, PWB, and coping strategies before and after neurosurgical procedures and to evaluate whether neurosurgical complications impact on these variables. This kind of study is useful to elucidate the relations between clinical and medical variables (such as complications, type of surgery, and radiological data) and sociodemographic variables with patient's reported outcome measures thus proving evidence that personal and environmental factors should be also considered for prognostic evaluation and treatment planning.

## 5. Conclusions

Our study showed that preoperatory period is particularly critical for patients undergoing neurosurgical procedures independently from the diagnosis. Consequently interventions that consider QoL, PWB, and psychological aspects should be considered for a more complete management of each patient. Future analysis will be performed also on postoperative QoL, PWB, and disability for evaluating the impact of neurosurgical procedures from the patient's subjective point of view.

## Figures and Tables

**Table 1:** Descriptive statistics of demographic and clinical variables.

Variable	Frequency	Percentage
Gender		
Male	103	48%
Female	95	52%
Age (years)		
Mean	51	
SD	13.5	
Range	19–83	
Educational level (years of study)		
Mean	12.4	
SD	4.25	
Disease		
CV	42	21.2%
ONC	89	45.0%
SD	54	27.3%
OS	7	3.5%
Other	6	3.0%

**Table 2:** Descriptive statistics of the 4 tests administered to the sample.

Test	Mean (SD)	Median, *Q*1–*Q*3	Min.–max.
KPS	95.71 (7.95)	100, 90–100	40–100
PGWB-S	61.76 (21.36)	62.3, 47.7–77	7.30–110.00
EUROHIS-QoL	3.4 (0.56)	3.38, 3.00–3.75	1.38–4.75
WHODAS-II	27.02 (18.14)	25, 11.11–38.89	0.00–83.33

**Table 3:** Comparisons between PGWB-S and EUROHIS-QoL and respective normative scores.

Test	Normative sample	*t*	*P*
PGWB-S	70.87	−6.000	<0.001
EUROHIS-QoL	3.68	−6.954	<0.001

**Table 4:** Coping strategies.

Coping strategies	6 or 7 (%)	8 (%)	Total
**BC_planning**	30.81	15.15	**45.96**
**BC_acceptance**	24.75	18.18	**42.93**
**BC_self-distraction**	28.79	11.11	**39.90**
**BC_positivereframing**	25.76	12.63	**38.38**
BC_behavioral disengagement	23.74	5.56	29.29
BC_active coping	21.21	6.57	27.78
BC_religion	15.66	9.09	24.75
BC_venting	17.17	4.55	21.72
BC_use of instrumental support	13.13	5.05	18.18
BC_humor	11.11	5.56	16.67
**BC_use of emotional support**	8.59	4.04	**12.63**
**BC_self-blame**	7.58	3.03	**10.61**
**BC_denial**	4.04	1.01	**5.05**
**BC_substanceabuse**	1.52	0.51	**2.02**

**Table 5:** Differences of test scores based on diagnosis, median (*Q*1–*Q*3).

Test	CV	ONC	SD	K − W	*P* value
KPS	100 (100-100)	100 (90–100)	100 (90–100)	7.728	0.021
PGWB-S	66.0 (54.07–77.0)	58.7 (44.0–77.0)	58.7 (51.3–80.7)	1.770	0.413
EUROHIS-QoL	3.5 (3.125–3.75)	3.37 (3.0–3.87)	3.37 (3.09–3.65)	0.730	0.694
WHODAS-II	19.44 (8.33–31.25)	22.2 (10.1–36.23)	33.3 (22.22–47.53)	14.315	0.001

**Table 6:** Relationship between gender, years of study, age, KPS, diagnosis, PGWB-S, WHODAS-II, and EUROHIS-QoL in the whole sample.

	*B*	SE	*β*	*P*
Constant	2.600	0.556		<0.001
Gender	0.000	0.075	0.000	0.998
Years of study	0.011	0.008	0.085	0.189
Age	−0.002	0.003	−0.058	0.365
KPS	0.004	0.005	0.052	0.441
CV versus ONC	−0.011	0.092	−0.008	0.909
SD versus ONC	−0.065	0.087	0.052	0.459
PGWB-S	0.010	0.002	0.383	<0.001
WHODAS-II	−0.009	0.002	−0.290	<0.001

**Table 7:** Relationship between gender, years of study, age, KPS, diagnosis, PGWB-S, WHODAS-II, and EUROHIS-QoL in CV, ONC, and SD groups.

CV	*B*	SE	*β*	*P*
Constant	3.606	1.418		0.016
Gender	0.120	0.175	0.108	0.498
Years of study	−0.001	0.020	−0.010	0.950
Age	−0.007	0.006	−0.168	0.279
KPS	−0.003	0.013	−0.039	0.798
PGWB-S	0.009	0.004	0.324	0.050
WHODAS-II	−0.012	0.005	−0.375	0.031

ONC	*B*	SE	*β*	*P*

Constant	2.837	0.796		0.001
Gender	−0.037	0.113	−0.029	0.744
Years of study	0.010	0.012	0.073	0.415
Age	−0.003	0.004	−0.065	0.450
KPS	0.003	0.007	0.035	0.707
PGWB-S	0.011	0.003	0.382	0.001
WHODAS-II	−0.011	0.004	−0.322	0.005

SD	*B*	SE	*β*	*P*

Constant	0.669	1.243		0.593
Gender	0.004	0.137	0.004	0.979
Years of study	0.006	0.018	0.047	0.758
Age	0.007	0.005	0.189	0.189
KPS	0.018	0.011	0.248	0.110
PGWB-S	0.009	0.004	0.395	0.020
WHODAS-II	−0.001	0.004	−0.023	0.890
